# Parental understanding and self-blame following sudden infant death: a mixed-methods study of bereaved parents' and professionals' experiences

**DOI:** 10.1136/bmjopen-2016-011323

**Published:** 2016-05-18

**Authors:** Joanna Garstang, Frances Griffiths, Peter Sidebotham

**Affiliations:** 1 Division of Mental Health and Wellbeing, University of Warwick, Coventry, UK; 2 Coventry and Warwickshire Partnership NHS Trust, Coventry, UK; 3 Division of Health Science, University of Warwick, Coventry, UK

**Keywords:** QUALITATIVE RESEARCH

## Abstract

**Objectives:**

Improvements in our understanding of the role of modifiable risk factors for sudden infant death syndrome (SIDS) mean that previous reassurance to parents that these deaths were unpreventable may no longer be appropriate. This study aimed to learn of bereaved parents' and healthcare professionals' experiences of understanding causes of death following detailed sudden unexpected death in infancy (SUDI) investigations. The research questions were: How do bereaved parents understand the cause of death and risk factors identified during detailed investigation following a sudden unexpected infant death? What is the association between bereaved parents' mental health and this understanding? What are healthcare professionals' experiences of sharing such information with families?

**Design:**

This was a mixed-methods study using a Framework Approach.

**Setting:**

Specialist paediatric services.

**Participants:**

Bereaved parents were recruited following detailed multiagency SUDI investigations; 21/113 eligible families and 27 professionals participated giving theoretical saturation of data.

**Data collection:**

We analysed case records from all agencies, interviewed professionals and invited parents to complete the Hospital Anxiety and Depression Scale (HADS) and questionnaires or in-depth interviews.

**Results:**

Nearly all bereaved parents were able to understand the cause of death and several SIDS parents had a good understanding of the relevant modifiable risk factors even when these related directly to their actions. Paediatricians worried that discussing risk factors with parents would result in parental self-blame and some deliberately avoided these discussions. Over half the families did not mention blame or blamed no one. The cause of death of the infants of these families varied. 3/21 mothers expressed overwhelming feelings of self-blame and had clinically significant scores on HADS.

**Conclusions:**

Bereaved parents want detailed information about their child's death. Our study suggests parents want health professionals to explain the role of risk factors in SIDS. We found no evidence that sharing this information is a direct cause of parental self-blame.

**Strengths and limitations of this study**

- This is a unique study because it looks at parents' explanations of causes of death within the context of new, detailed child death investigations.
- This study allowed for a detailed understanding of cases due to the triangulation of data from parental interviews, professional interviews, questionnaires and case records.
- The use of a validated mental health screening tool (Hospital Anxiety and Depression Scale, HADS) enabled the role of anxiety and depression to be considered in relation to parental self-blame.
- The study was limited by low recruitment, but despite this, a wide diversity of parental and professional experiences were captured.

## Introduction

The sudden death of an infant is one of the most devastating events that can happen to parents and the pain is still felt many years later.^1^ Only a minority of sudden unexpected death in infancy (SUDI) cases have a complete medical explanation for death; most remain unexplained and are often labelled as sudden infant death syndrome (SIDS), which is a diagnostic term used when despite detailed investigation no cause for death can be determined.^2^ Bereaved parents often blame themselves and feel guilty for these deaths due to the lack of explanation for them^3–6^ but self-blame is also a common feature of grief.^7^ Prior to our current understanding of SIDS, recommended practice for healthcare professionals was to reassure SIDS parents that their actions played no role in the death as SIDS was neither predictable nor preventable; it was perceived this would help alleviate the parents' feelings of self-blame.^8^ ^9^ SIDS, however, can be understood as a complex interplay between intrinsic vulnerability, a critical period of homoeostatic development, and exogenous stressors.^10^ ^11^ The exact pathophysiological pathways leading to SIDS and how identified exogenous stressors lead to death remain unknown, but there is extremely strong epidemiological evidence around these stressors. A recent review^12^ highlighted the major modifiable risk factors for SIDS as non-supine sleeping, parental smoking, head covering, use of soft bedding and co-sleeping on a sofa or with parents who have consumed alcohol or smoke. With this knowledge, SIDS could be considered to be partly related to parental actions and choices; therefore, the previous explanations and reassurances given to parents may no longer be appropriate.

In many countries, including the UK, there are now detailed investigations following unexpected child deaths aiming to learn the full cause, including modifiable risk factors, for all deaths.^13^ In the UK, this is known as the joint agency approach (JAA) or ‘rapid response’. As part of this approach, healthcare professionals are expected to share all information about the death with bereaved families.^14^ Previous research has shown the importance for parents of understanding the reasons why their child died after a sudden child death,^15^ but there has been only very limited research on how parents understand modifiable risk factors after a SIDS death. One study of SIDS in families with substance abuse highlighted that modifiable risk factors can be interpreted as causal mechanisms increasing parental feelings of guilt.^16^ Given that previous practice was to reassure parents to prevent self-blame, there is the possibility that sharing detailed information with parents could be harmful, potentially inducing self-blame and guilt which are associated with more severe and prolonged grief reactions.^7^ ^17^

We studied bereaved parents' understanding of why their infant died and their reactions to this, along with professionals' experiences, as part of a mixed-methods evaluation of the new UK JAA to investigating SUDI. This enabled us to learn of parents' and professionals' experiences in the new context of sharing information for SIDS as well as for medical causes of SUDI.

The research questions were:

How do bereaved parents understand the cause of death and risk factors identified during detailed investigation following a sudden unexpected infant death?
What is the association between bereaved parents' mental health and their understanding of cause of death and risk factors following a sudden unexpected infant death?
What are healthcare professionals' experiences of sharing such information with families?

## Methods

### Inclusion and exclusion criteria

SUDI is defined as the death of an infant which was not anticipated as a significant possibility 24 h before the death or where there was a similarly unexpected collapse leading to or precipitating the events which led to the death.^18^ Parents of SUDI cases were eligible for the study regardless of the final cause of death providing that infants had lived and died in the counties of Herefordshire, Shropshire, Staffordshire, Warwickshire, West Midlands and Worcestershire; were aged between 1 week and 1 year at death; and died between 1 September 2010 and 31 August 2013. The JAA investigation had to be completed prior to contacting parents; cases with ongoing criminal investigations were excluded.

### Identification and recruitment of cases

We were notified of all eligible SUDI cases by the departments of pathology at Birmingham Women's Hospital and Birmingham Children's Hospital; all SUDI postmortem examinations for the region are performed at these centres. We then contacted the local paediatrician responsible for managing the JAA investigation for each case and asked them to inform families of the study, or to inform us of the reasons if this was not possible. This took place during follow-up appointments after the JAA investigations had concluded and was usually 6–12 months after the death. Interested parents agreed for their contact details to be passed to the lead author who telephoned them with further information.

### Data collection

#### In-depth parental interviews and questionnaires

Our original intention had been to conduct a survey of parents' experiences of JAA investigations following SUDI by means of a face-to-face structured interview, and seek parents' permission for us to access case records from all agencies relating to their JAA. The survey results were to be used for purposive sampling for in-depth interviews with parents and professionals. However, due to difficulties with recruitment, and many parents' desire to give full accounts of their experiences, 10 months into the study we revised this method and offered all subsequent parents a choice of having an initial in-depth interview or completing a postal questionnaire, in addition to case record analysis. We asked all parents who had completed an interview or questionnaire to take part in a follow-up interview around 2 years after the death; due to time constraints, this was only possible for cases dying in the first 2 years of the study.

The lead author visited parents at home (or location of their choice) to conduct the in-depth interview or structured questionnaire-based interviews; these lasted between 1 and 4 h. The lead author was a female paediatrician experienced in JAA investigations and a PhD student, this was explained to participants. In-depth interviews were audio-recorded and transcribed in full, field notes were made for all interviews. The field notes detailed parents' demeanour, the location of the interview, aspects of the interview parents found difficult to answer, and presence of other family members. Both in-depth interviews and questionnaires covered the parents' experiences of the JAA investigation from the time they found their lifeless baby until the final contact with professionals concerning the death. Parents were asked to describe the events leading up to the death; to explain their understanding of why their baby died, this included a description of the cause of death as well as whether parents thought that they had understood this cause; how the cause of death had been explained to them; and their health after the death. Parents were not specifically asked about risk factors but these often arose as part of the conversation concerning the cause of death. The follow-up interview covered the same issues, and parents were asked if they had had any further thoughts about the death or the JAA since the first interview. The postal questionnaire covered an identical range of topics as the questionnaire-based interview, but parents gave less detailed answers.

The interviewer had no knowledge of any details pertaining to cases prior to interviewing parents, so she could learn of parents' accounts and their explanation of the cause of death without preconceptions. However, by the time of follow-up interviews, the interviewer had studied case records, so was aware of causes and risk factors for deaths and could sensitively probe parents about these if necessary. Parents were not specifically asked about issues of blame, but this topic frequently arose spontaneously during both initial and follow-up interviews particularly when parents explained about sleep situations, causes of death and explanations of risk factors by professionals.

#### Assessment of parental mental health

As mental health problems are common following the death of an infant,^19^ potentially affecting the parents' perception of events, and negative experiences of investigations following child death may be associated with subsequent mental health problems, we asked all parents to complete the Hospital Anxiety and Depression Scale (HADS). This consists of seven questions relating to depression and seven to anxiety. In both domains, a score of 8–10 is of borderline significance and a score of more than 11 is considered clinically significant; the maximum score is 21.^20^

#### In-depth professional interviews

For the cases where parents had initial in-depth interviews, the lead author aimed to interview the police officers, paediatricians, specialist nurses and social workers who had taken part in the JAA investigation; these were conducted after the parental interviews. The professional interviews took place either in person or by telephone; they were audio-recorded and transcribed in full. Interviews lasted between 20 min and 1 h. Professionals had access to the case records, so they did not have to rely on recall alone but usually they reported that they vividly remembered the case. Professionals were asked about their experiences of JAA investigations and about discussions regarding cause or risk factors for death relating both to the recruited case and more generally.

#### Case records

We studied the coroner's inquisition, postmortem examination report, JAA final case discussion (FCD) notes and a summary of the Child Death Overview Panel review (form C) for each case for details of cause of death and for any mention of risk factors for SIDS cases. Data were extracted from these using standard proformae to ensure the same information was collected in each case.

#### Social deprivation scores

We obtained the Income Deprivation Affecting Children Index (IDACI)^21^ scores and ranks for all SUDI cases regardless or recruitment status; these were provided for us by the pathology department without disclosing any patient identifying information. This allowed us to compare social deprivation between recruited and non-recruited cases, so that we could assess if the recruited sample was representative of SUDI cases more generally.

### Data analysis

We split the cases into two different groups: one for medically explained deaths and the other for SIDS; this allowed us to compare parental understanding and other themes between the two groups.

#### Questionnaire and interview data analysis

The qualitative data consisted of free-text comments from questionnaires, in-depth interview transcripts and field notes; we analysed these data using a Framework Approach^22^ with NVIVO 10 software. Framework Approach was developed for policy evaluations; it is grounded in the original accounts of the participants of research, is a systematic process and allows for within-case and between-case analyses. We checked all transcripts for accuracy with the audio-recording. We then coded all in-depth interview data, field notes and free-text comments from questionnaires. The codes were not determined in advanced but developed as transcripts were coded. After coding 10 parental interviews, the codes were summarised and discussed with the whole research team (all authors of this paper) and refined or merged when needed. The coding was also reviewed with the project steering group consisting of SUDI professionals from all agencies and bereaved parents. The coding structure was then applied to the professional interviews. Data analysis started while data collection was ongoing.

The codes were finally inductively categorised into three main themes: understanding the cause of death, experiences of the JAA and well-being (the latter two are reported elsewhere). Within the theme understanding the cause of death, there were four subthemes: needing answers, cause of death, understanding risk factors and blame. Data relating to parental blame was categorised as self-blame, blaming others, feeling blamed and blaming no-one. We returned to the data to look at parental interview transcripts as a whole for any themes that were present throughout each interview. We identified parental interviews where overwhelming parental self-blame was apparent.

We managed the data from follow-up interviews identically to the data from initial interviews. The parents' description of causes for death and risk factors was very similar at both time points, and no additional codes were needed when analysing the data from follow-up interviews.

#### Statistical analysis

Using SPSS, we performed independent t tests comparing IDACI scores and ranks for recruited and non-recruited cases. We also performed independent t tests comparing HADS between parents who expressed high levels of self-blame for the death and those who did not.

### Integration of data

We created a framework matrix using NVIVO for each case with data for each code relating to understanding the cause of death from the parental interview or questionnaire, professionals' interviews, case records, and with the HADS. The parents' descriptions of the cause of death were compared with the cause of death as given by the JAA FCD. We took the cause of death from the FCD to be the most comprehensive as it is the consensus of professional opinion taking into account findings from reviewing the scene of death, infant medical and social histories, and postmortem examinations. We categorised any death remaining unexplained at FCD as SIDS.

### Ethical issues

We had safeguards in place to protect the bereaved families from feeling pressured to participate. The initial contact was made through local paediatricians. If parents agreed to be contacted about the study, there was a 2-week ‘cooling-off’ period before the research team telephoned. Similarly, interview dates were arranged at least 2 weeks ahead to avoid rushing parents into decisions about participation.

At the start of interviews, parents were told that they could stop the interview or withdraw from the study at any point during the interview or subsequently. At the end of the interview, parents were given an information sheet with details of the Lullaby Trust, a support organisation for bereaved parents, so they could access these services if needed. In some instances, parents appeared to have significant mental health issues; in these cases, parents were encouraged to contact their general practitioner. As part of the informed consent process, parents were told that if they disclosed information that could lead to concerns about child abuse further action would need to be taken including possibly referring the matter to police and social care. Such action was taken with one family.

## Results

### Recruitment and participants

There were 113 SUDI cases, 9 were excluded due to ongoing investigations leaving 104 eligible SUDI cases of which 23 (22%) were recruited to the study. In 32 (28%) cases, paediatricians did not inform parents of the study; the reasons for this are largely unknown due to these paediatricians not responding to our emails or telephone calls, but some expressed worries about distressing families further by mentioning the study. In 29 (26%) cases, parents were asked but declined to participate, and in 20 (18%) cases, parents did not attend follow-up appointments with paediatricians. There was no significant difference in social deprivation between recruited and non-recruited cases using the IDACI^21^ (data were only available for 88/90 non-recruited cases); these are shown in table 1.

**Table 1:** Comparison of Income Deprivation Affecting Children Index (IDACI) scores and ranks between recruited and non-recruited sudden unexpected death in infancy cases

	Recruited cases N=23	Non-recruited cases N=88	Independent t test
Mean (95% CI) IDACI score	0.314 (0.232 to 0.395)	0.367 (0.328 to 0.406)	t (109)=−1.21 p=0.229
Median IDACI rank	6702	5134	t (109)=0.654 p=0.514
Mean (95% CI) IDACI rank	9206 (5617 to 12 796)	8012 (6419 to 9605)	

The results in this paper are based on the 21/23 recruited families having interviews or completing questionnaires; those opting for case note analysis alone were excluded from this part of the study. The details of their participation are shown in table 2.

**Table 2:** Details of participating families

	SIDS	Medically explained deaths	Total SUDI cases	Mothers and fathers participating jointly	Mothers participating alone*
Initial in-depth interview only	8	3	11	6	5†
Structured questionnaire-based interview only	2	1	3	2	1
Postal questionnaire only	0	1	1	1	0
Structured questionnaire-based interview and in-depth follow-up interview	3	1	4	3	1
Initial and follow-up in-depth interviews	1	1	2	1	1
Total	14	7	21	13	8

*No fathers participated alone.

†One mother was supported by her own mother and one by a friend for the interview.

SIDS, sudden infant death syndrome; SUDI, sudden unexpected death in infancy.

Interviews took place a mean of 27 weeks (range 20–44 weeks) after the death for structured interviews, 50 weeks (range 36–80 weeks) for in-depth interviews and 26 months (range 24–28 months) for follow-up interviews.

In-depth interviews with professionals were held in 12/14 cases that had initial in-depth interviews; these involved 14 police officers, 10 paediatricians, two specialist nurses and two social workers. (One police officer and two paediatricians were interviewed twice about two separate cases.) In the remaining two cases, no relevant professional could be traced due to retirements and relocations.

We obtained theoretical saturation of data meaning that no new data were emerging during interviews that were relevant to the issues under investigation;^23^ these issues were of self-blame and parents needing to know the cause of death. These themes were not identified in advance but emerged during the data analysis as topics of importance to the parents. Three further families were interviewed after theoretical saturation was obtained; no new themes emerged from these interviews.

The term ‘parents’ is used for either mothers or fathers. Within couples, parents tended to have similar explanations for the death and understanding of risk factors, but where findings are gender-specific, the terms ‘mother’ or ‘father’ are used instead. Quotes are not attributed to individual cases to preserve anonymity.

### Description of cases

The mean age at death of recruited cases was 100 days (95% CI 69 to 131 days). In 14 cases, the death remained unexplained and was categorised as SIDS. Seven deaths were due to fully explained medical causes such as previously undetected congenital heart disease or overwhelming infection.

### Risk factors

**Figure 1:**
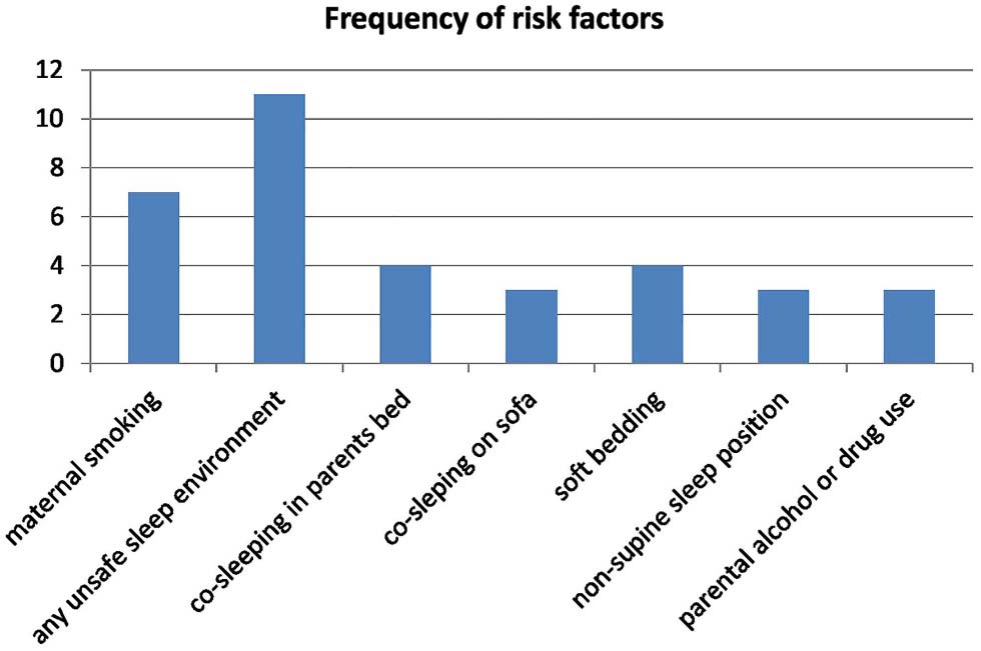
Frequency of risk factors in sudden infant death syndrome infants.

Multiple modifiable risk factors were recorded in case records for all but one SIDS death; the frequency of the most common risk factors identified is shown in figure 1. Five SIDS infants were recorded as being intrinsically vulnerable due to multiple birth, prematurity, family history or congenital anomalies.

## Key findings

### Parents have a strong need to know the cause of death

The parents' need to understand why their baby died came across very strongly in most interviews despite parents not being asked specifically about this. This was the case for explained deaths and SIDS (quote 3.1). There was usually a wait of at least 4 months for the postmortem examination results due to histology and metabolic tests; parents often became increasingly anxious about the cause of death during this time (quote 3.2). Parents were relieved if there was a medical explanation for the death and professionals commented on the comfort and benefit this brought to parents (quote 3.3). Conversely, some SIDS parents felt cheated by the lack of complete explanation for their infants' deaths increasing their distress (quote 3.4). Example quotes are shown in table 3.

**Table 3:** Parental quotes on understanding the cause of death

Theme	Reference number	Quote
Needing to know cause of death	3.1	Yes, I suppose I felt it was quite important really to hear what the findings were really because it was unexpected, she was such a healthy girl and it was such a shock…I really wanted to know and that was all really I guess. (father, SIDS case)
Anxiety while waiting for results	3.2	That's what you…you turn it on yourself when you don't hear anything, then you make things up in your head. ‘It must have been this, it must have been this, it must have been this’…because you don't know anything…Which leaves me to sit there wondering what it was and thinking ‘we don't know anything about the toxicology’ and I'm thinking ‘how could you possibly have poisoned…how have you poisoned him?’ Well you don't know, until that comes back, you don't know, and that was weeks. (mother, SIDS case)
Comfort from knowing the cause of death	3.3	…for me that was amazing, seeing her [the mother] the week after [explaining the cause of death] because she was just a totally different woman. This was a woman that didn't go outside, never smiled and she was up, she was dressed, she was, you know, smiling…a totally, totally different woman from when we first saw her, it was just amazing, just the results of that just changed her completely. (nurse, medically explained death)
Distress at no explanation for the death	3.4	It's just, not having an answer; I don't think it's fair like…why? (mother, SIDS case)

SIDS, sudden infant death syndrome.

### Parents' explanation for medical causes of death

Parents of six infants with medical causes for death felt they understood the cause and gave descriptions which closely matched the causes of death in the FCD documents. One mother said that she did not understand the cause of death at all and did not attempt to describe it.

### Parents' explanation for SIDS

Parents of eight SIDS infants gave accounts that showed that they grasped the concept that deaths could be fully investigated but no cause of death established (quote 4.1). Some parents also described vividly how they thought their babies had died (quote 4.2); these lay accounts fit well with current physiological understandings of SIDS.^24^ Others still questioned how SIDS could be a natural process (quote 4.3) despite having received detailed explanations of SIDS from paediatricians. Two SIDS families had some comprehension of the concept of unexplained deaths but struggled with the idea that there could be pathological findings which were insufficient to explain the death (quote 4.4). Parents of the four remaining SIDS infants felt they had understood the cause of death; however, their explanations differed significantly from those in the FCD documents. One mother explained that her baby had died of ‘bronchitis’ when the cause from FCD was of SIDS with minor lung pathology. The three other cases overlap significantly with understanding modifiable risk factors as these parents made no mention of unsafe sleep environments in their explanations; these cases will be described in the next section. Example quotes are shown in table 4.

**Table 4:** Quotes concerning parental understanding of cause and risk factors for death

Theme	Reference number	Quote
Understanding SIDS	4.1	…that was one of the things I asked the paediatrician, I said ‘what is it?’ and she said ‘that's the whole point, we don't know’. (mother, SIDS case)
	4.2	…something in his brain…he'd stopped breathing and his brain wasn't developed enough to sort of say…‘baby, you're not breathing, breathe son’. (father, SIDS case)
How can SIDS be natural?	4.3	I know they are saying natural causes but what's natural about a healthy person dying? (mother, SIDS case)
Incomplete explanations for death	4.4	The paediatrician said that the baby had had some bleeding and not just at the time of death,…she'd had previous bleeding that had resolved itself…And yet he said the people who did the autopsy couldn't see how that would have caused her to die. (mother, SIDS case)

SIDS, sudden infant death syndrome.

### SIDS parents' understanding of modifiable risk factors

The parents of six SIDS infants appeared to understand the relevance of risk factors for their infants' deaths. These parents talked openly about the unsafe sleep environments or parental smoking that their babies had been exposed to (quote 5.1) or had discussed these with health professionals (quote 5.2). One parent even described their child's death using the triple risk hypothesis^10^ (quote 5.3). For other parents, it was clear that the original discussion of risk factors with the paediatrician had been difficult, and it was similarly difficult for them to discuss these during the interview. The dilemma for parents is that by acknowledging modifiable risk factors for the death, parents are acknowledging that, had they made different choices, their babies may not have died; thus, this understanding may increase the parents' pain (quote 5.4). Some parents' description of the role of risk factors varied throughout the interview; at times accepting risk factors as relevant and at others downplaying them by portraying deaths as inevitable, deeming risk factors irrelevant (quote 5.5). This fluctuation in acceptance could be seen as a way of coping with the pain of realising that the death may have been preventable. The parents of three SIDS infants made no mention of relevant risk factors despite evidence of discussions about these between them and paediatricians. For example, a parent said that their baby had died of ‘straightforward SIDS’ which was not further elaborated although the case records and professional interviews detailed their explanations to the parent of the role that alcohol, drug consumption and co-sleeping may have played in the death. It was not clear from the interviews whether the parents were minimising the significance of the risk factors, denying them completely to protect themselves from the reality of the knowledge, or that they simply did not understand despite the explanations given to them. These parents only had initial interviews, so the interviewer had no access to case records beforehand, so was unable to discuss with the parent how they had reached their own explanations.

Paediatricians and specialist nurses were asked during their interviews about their explanation of risk factors to parents. Five paediatricians and one specialist nurse commented that they found this very difficult and worried that it would lead to the parents self-blaming. Two paediatricians said that they avoiding discussing risk factors because of the risk of self-blame (quote 5.6) or that these discussions should be left until the next pregnancy and managed by specialist services for families with infants born following SIDS. Example quotes for this theme are in table 5.

**Table 5:** Quotes concerning understanding risk factors

Theme	Reference number	Quote
Understanding risk factors	5.1	If they say that nine out of ten cot deaths are in families where family members smoke, whether you do it around the baby or not…But I was thinking to myself ‘I can't see how that makes any difference’ and I mean, I fell asleep with him by accident that night when it happened but the amount of times I'd put him in bed with me…(mother, SIDS case)
	5.2	She clearly understands and I mean she did say to me when she was pregnant with the [next] baby…she said ‘I'm going to be really, really, really clear this time, that this baby will be sleeping in their own crib and that as much as I might be tempted, I will not be co-sleeping’. (nurse, SIDS case)
	5.3	Basically that…pretty much what that Prof Kinney says, he must have been a vulnerable baby put into an unsafe environment. (mother, SIDS case)
Pain of acknowledging actions	5.4	Yes because my wife sort of listened to it [the paediatrician talking about risk factors] and thought ‘well he was in our bed at the time when he died and should I have put him in there…had I put him in his cot, would things have turned out differently?’ (father, SIDS case)
Understanding and downplaying	5.5	In a way it's made me open my eyes a lot more as well because you don't realise what it could do like with co-sleeping but I weren't actually right next to him like I usually…I can understand what they are saying about it…obviously he didn't want me to wake up next to him, he knew [he was going to die]. That's how I've got to look at it. (mother, SIDS case)[baby found in co-sleeping situation in corner of small sofa with head covered by bedding, baby normally slept next to mother on this sofa]
Fear of parents self-blaming when risk factors discussed	5.6	…So once the death has happened, we don't…I don't think we dwell on the risk factors because I think, that's right, we're not trying…we don't want to apportion blame to parents. (paediatrician, SIDS case)

SIDS, sudden infant death syndrome.

### Blame

Parents were not asked specifically about blame during interviews, but this topic often came up spontaneously. We identified four different categories of blame relating to parents: self-blame, blaming others, feeling blamed and blaming no-one. The category of blame seemed unrelated to cause of death (medically explained or SIDS) and of SIDS parents' understanding of modifiable risk factors. Many parents expressed more than one category of blame. Parents concurred with their expressions of blame with the notable exception of self-blame which occurred nearly always in mothers.

Six mothers and one father (relating to six cases) said that they blamed themselves for the death either partially or completely; all six mothers described feeling guilty because their baby had died; they had failed in their role as a mother (quote 7.1); these feelings of guilt did not relate to the cause of death (quote 7.2). Three of these cases were of SIDS in unsafe sleep environments, and three were medically explained deaths which were unpreventable.

Three mothers said they blamed themselves completely for the death and had feelings of overwhelming guilt; two of the infants died of medical causes and one of SIDS. Notably, all three mothers scored highly for anxiety and depression on HADS with all scores in the clinically significant range; none of the other mothers had clinically significant scores, these are shown in table 6.

**Table 6:** Hospital Anxiety and Depression Scale (HADS) scores of self-blaming and non-self-blaming mothers

	Mean (95% CI) HADS anxiety score	Independent t test for HADS anxiety score	Mean (95% CI) HADS depression score	Independent t test for HADS depression score
Overwhelming self-blame (n=3)	17.0 (14.5 to 19.5)	t (19)=−3.91, p<0.001	18.3 (15.5 to 21.2)	t (19)=−3.68, p<0.002
Moderate or no self-blame (n=18)	9.9 (8.4 to 11.5)		8.8 (6.6 to 11.0)	

In three cases (two SIDS, one medical death), the parents felt blamed by the professionals for the death. Regarding the SIDS cases, one mother felt very blamed by the paediatrician who explained about the unsafe sleep situation in which the infant had died. However, at a follow-up interview 2 years later, the mother no longer felt blamed and could barely recall her animosity towards the paediatrician. Another SIDS mother felt blamed by the police while she was in the emergency department; maternal alcohol consumption and illicit drug use may have been implicated in the death but the mother did not refer to this. The parents of an infant dying of a medical condition felt blamed by the doctors in the emergency department; analysis of their account suggests that poor communication may have been the cause for this.

The parents of five infants (two SIDS, three medical deaths) blamed other people for the deaths; for some, this may have been a deflection from acknowledging their own actions. For example, one couple spoke at length about a perceived lack of care from their general practitioner a few days prior to the death (an examination of the case record did not suggest any lack of care) but in the same interview also described their own inaction in the face of the baby's deteriorating condition. However, in other cases, parental blame seems justified to some extent; for example, one infant died shortly after discharge home from hospital despite having had a witnessed unexplained respiratory arrest only a few days previously. Conversely, other parents did not seek to blame others when they could have had good reason to do so; these parents recalled inappropriate advice given to them by healthcare staff which may have been a factor in the death (quote 7.3).

In six cases, parents made no mention of blame at all, and in six other cases, parents explained that they blamed no-one for the death; again there was no relationship with the cause of death or parental understanding of modifiable risk factors. Some mothers had initially blamed themselves for the death, but as time passed no longer did so. In other cases where modifiable risk factors were relevant, parents accepted responsibility for their choice of actions but not blame; viewing this as a negative option (quote 7.4). However, other SIDS parents suggested that despite the presence of modifiable risk factors, death could not have been prevented or that their actions did not have a bearing on the death; they interpreted the label of SIDS as an absolution, so that there could be no blame attributed (quote 7.5). In these cases, a lack of self-blame may be part of a self-protection mechanism and could almost be a denial of the issues surrounding the death. Example quotes for blame are in table 7.

**Table 7:** Parental quotes on blame

Theme	Reference number	Quote
Maternal blame	7.1	At this point I didn't have any idea how long I'd been asleep and then feeling this overwhelming guilt…I've slept for hours and she's just died. (mother, medically explained death)
	7.2	I blame myself, if I hadn't have gone back to work, he'd be fine. (mother, medically explained death)
Not blaming others	7.3	We're not trying to put fault on anybody, it could be anything still but they should have clear guidelines, shouldn't they? (father, SIDS case)
Responsibility not blame	7.4	And I could choose to let myself feel very guilty and that in a sense would kill your spirit…I'm happy to accept that I have some responsibility in his death and that's a different thing to being guilty. (mother, SIDS case)
Absolution of SIDS	7.5	We've both always said we were quite glad when it came back that it was Sudden Infant Death…because it's been Sudden Infant Death, we sort of go ‘well we couldn't have done anything, if it was going to happen, it was going to happen…(father, SIDS case)

SIDS, sudden infant death syndrome.

## Discussion

Our results suggest that most parents really want to know why their baby died; not knowing why their baby died may cause further distress to parents, whether this is due to long waits for the results of postmortem examinations or because deaths remain unexplained. An unexplained death by its nature is an unpredictable event rendering the parents powerless to prevent future tragedies, thus increasing the anxiety and grief;^25^ having as much information as possible should help parents to emotionally accept and make sense of the death to themselves. In this study, most SIDS parents seemed to understand the concept that these deaths remain unexplained after full investigations and that modifiable risk factors may play a role; however, paediatricians were sometimes reluctant to share this information with parents, fearing it will lead them to blame themselves. Self-blame was common in mothers following both medically explained deaths and SIDS; however, when overwhelming, it appeared to be associated with clinically significant anxiety and depression. Self-blame was not associated with the cause of death, presence of modifiable risk factors or parental understanding of these.

This study allowed for a detailed understanding of cases due to the triangulation of data within each case from parental interviews, professional interviews, questionnaires and case records from every agency. This was vital to our study of SIDS parents' understanding of risk factors. For example, some SIDS parents made no mention of risk factors at interview, and without access to these other data sources, we would not have known whether professionals had discussed risk factors with them or not. We obtained more detailed data from in-depth interviews compared with questionnaires particularly concerning issues of blame, but the parents' need to know why their baby died and their understanding of the cause of death came across strongly in questionnaires and in-depth interviews. As with all qualitative research, limitations include that few people participate, results may be highly subjective, and therefore difficult to generalise. We did indeed struggle to recruit bereaved families. However, despite the limited recruitment, we captured a wide diversity of parental and professional experiences, and the recruited cases were from socially diverse backgrounds with similar levels of social deprivation when compared with all SUDI cases in the region. Given the diversity of experiences, theoretical saturation of data and a rigorous approach to data analysis, the findings of this study are likely to be relevant to the management of sudden infant deaths in other locations with similar detailed investigative processes and cultural backgrounds. Although the HADS scores were significantly different between mothers exhibiting overwhelming self-blame and mothers who did not, the sample size was small, thus limiting the reliability of this finding which will need confirmation from a larger study. We interviewed many parents together as couples; these parents did not have the opportunity to give their own individual account. It is possible that some parents (particularly fathers) may not have fully shared their feelings with us trying to protect their partners from further distress or that only the dominant parent's view was voiced.

This is a unique study because it looks at parents' explanations of causes of death within the context of new, detailed child death investigations and sharing of information about modifiable factors in deaths with parents. Although there have been other evaluations of these child death investigations,^26^ ^27^ to date there has been only limited research on parental experiences of these; a recent US study found that following detailed medicolegal infant death investigations, 29% of families never received any information on the cause of death, and for most of those who did so, this was by telephone only.^28^ Previous studies of parental guilt following infant death were conducted prior to knowledge of risk factors for SIDS when professional practice was typically to inform parents that their actions played no role in the death;^5^ ^6^ ^17^ these findings may be less valid with current understanding of SIDS. Our findings that parents want full information about the death concur with a recent systematic review of bereaved parents' wishes.^15^

Ideally, SIDS parents should be informed of and helped to understand the relevant modifiable risk factors; without this, their explanation for the death is incomplete. As our understanding of SIDS has changed, we should consider changing our explanations to parents; the view that we should keep potentially upsetting information from parents to avoid them self-blaming may risk greater harm. Parents may find out this information for themselves and have no supportive professional to discuss it with; or they may not learn of modifiable risk factors at all and expose subsequently born children to the same risks. Arguably, these discussions could be left for specialist support services for subsequently born infants; however, not all families will choose to partake in such services, and in the UK Care of Next Infant (CONI) scheme, there have been several deaths in unsafe sleep environments reported.^29^

Sharing such information on extrinsic risk factors for SIDS is based on the assumption that these are modifiable but this is not necessarily so. Parental actions such as alcohol consumption or smoking are often rooted in social deprivation so are less amenable to change; as illustrated by data from New Zealand where SIDS rates remains much higher in the socially deprived Māori population than in those of European descent despite public health campaigns.^30^ Parental behaviours such as co-sleeping are often based on long-standing cultural practices, facilitating breast feeding as well as enhancing parental and infant sleep; co-sleeping may in some cases be an entrenched behaviour and not easily changeable.^31^ Given that SIDS is a rare event, mothers may accept the risks of unsafe sleep, if this results in better quality sleep for themselves and their babies.^32^ In our study, some mothers unintentionally co-slept on sofas; these deaths could be seen as avoidable parenting errors or the inevitable consequence of exhausted, unsupported mothers. We should acknowledge that risk factors may not be easily modifiable, but this should not stop us sharing the information with parents, to help them understand more about why their child died and to assist them in making informed choices with subsequent infants.

Although we would aim to share full information on potentially modifiable risk factors for SIDS with bereaved parents, we do not want them to blame themselves; the parents' actions were carried out with no intention of causing harm. Some self-blame may occur with detailed understanding of deaths; this is unsurprising given that public perception is that most accidents are preventable.^33^ Overwhelming maternal self-blame in this study did not appear to be related to the cause of death or understanding of risk factors but may have been related instead to symptomatic anxiety and depression. Self-blame can be a normal part of grieving after infant death: by blaming oneself for the death, it stops being a random, unexplained event, and can be controlled, giving a sense of order; this situation may be easier to live with.^25^ In contrast, when blaming other persons, this is more of a value judgement: either following a logical stepwise process^34^ or following constant re-evaluations in the light of new evidence.^35^ A high level of self-blame in bereaved adults is associated with higher levels of depression, more severe grief reactions and slower recovery from grief.^7^ A similar association between self-blame, anxiety and depression has also been seen in mothers of stillborn infants.^36^ Self-blame and increased intensity of grieving following perinatal death also relate to some extent to pre-existing personality traits.^37^ ^38^ Our results suggest that parents want to know why their infants died and that they can understand the role of risk factors in SIDS. Our findings should provide reassurance that sharing of detailed information by healthcare professionals is what parents want. We found no evidence that sharing this information is a direct cause of parental self-blame.
